# Supplementary material for: Modelling Skylarks (Alauda arvensis) to Predict Impacts of Changes in Land Management and Policy: Development and Testing of an Agent-Based Model
Source: PLoS One. 2013 Jun 6;8(6):e65803. doi: 10.1371/journal.pone.0065803 (PMC3675089; doi:10.1371/journal.pone.0065803)
Supplement: Supporting Information S4 — The skylark ODdox as a zipped archive. (ZIP) [file pone.0065803.s004.zip › Skylark_ODdox/class_skylark___male.html]

ALMaSS Skylark ODdox: Skylark\_Male Class Reference


ALMaSS Skylark ODdox
 2.0


- Main Page
- Related Pages
- Classes
- Files

- Class List
- Class Index
- Class Hierarchy
- Class Members

Public Member Functions |
Public Attributes |
Protected Member Functions |
Protected Attributes

Skylark\_Male Class Reference

`#include <skylarks_all.h>`

List of all members.

|  |  |
| --- | --- |
| Public Member Functions | |
| virtual void | BeginStep (void) |
| int | DefendTerritory () |
| virtual void | EndStep (void) |
| void | OnAddNestling (Skylark\_Nestling \*N) |
| void | OnAddPreFledgeling (Skylark\_PreFledgeling \*P, Skylark\_Nestling \*N) |
| void | OnBroodDeath () |
| void | OnBroodDesertion () |
| void | OnEggHatch () |
| bool | OnEvicted () |
| double | OnFoodMessage (int n, double f, double p) |
| void | OnMateDying () |
| void | OnMateLeaving () |
| void | OnMateNeverComesBack (Skylark\_Female \*AFemale) |
| void | OnNestlingDeath (Skylark\_Nestling \*N) |
| void | OnNestLocation (int x, int y) |
| void | OnNestPredatation () |
| void | OnPairing (Skylark\_Female \*female) |
| void | OnPreFledgelingDeath (Skylark\_PreFledgeling \*P) |
| void | OnPreFledgelingMature (Skylark\_PreFledgeling \*P) |
| void | OnReHouse () |
| void | SensibleCopy () |
|  | Skylark\_Male (int x, int y, double size, int age, SkTerritories \*Terrs, Landscape \*L, Skylark\_Population\_Manager \*SPM, int bx, int by, int mh) |
| virtual void | Step (void) |
| skTerritory\_struct | Supply\_Territory () |
| double | Supply\_TerritoryQual () |
| int | SupplyBroodAge (int n) |
| int | SupplyBroodSize () |
| int | SupplyBroodWeight (int n) |
| Point | SupplyNestLoc () |
| bool | SupplyNestValid () |
| int | SupplyNoHabitatRefs () |
|  | ~Skylark\_Male () |
| Public Member Functions inherited from Skylark\_Adult | |
| virtual void | CopyMyself (int a\_sktype) |
|  | Skylark\_Adult (int x, int y, double size, int age, SkTerritories \*Terrs, Landscape \*L, Skylark\_Population\_Manager \*SPM, int bx, int by, int mh) |
|  | ~Skylark\_Adult () |
| Public Member Functions inherited from Skylark\_Base | |
| void | AddStriglingMort (int lifestage) |
| bool | InSquare (rectangle R) |
| virtual double | On\_FoodSupply (double) |
|  | Skylark\_Base (int x, int y, SkTerritories \*Terrs, Landscape \*L, Skylark\_Population\_Manager \*SPM, int bx, int by, int mh) |
| virtual int | WhatState () |
| Public Member Functions inherited from TAnimal | |
| void | CheckManagement (void) |
| void | CheckManagementXY (int x, int y) |
| virtual void | CopyMyself () |
| virtual void | Dying () |
| virtual void | KillThis () |
| void | SetX (int a\_x) |
| void | SetY (int a\_y) |
| int | Supply\_m\_Location\_x () |
| int | Supply\_m\_Location\_y () |
| unsigned | SupplyFarmOwnerRef () |
| int | SupplyPolygonRef () |
| AnimalPosition | SupplyPosition () |
|  | TAnimal (int x, int y, Landscape \*L) |
| Public Member Functions inherited from TALMaSSObject | |
| void | OnArrayBoundsError () |
|  | TALMaSSObject () |
| virtual | ~TALMaSSObject () |

|  |  |
| --- | --- |
| Public Attributes | |
| int | BroodAge |
| bool | HaveTerritory |
| vector< int > | m\_HabitatTable\_PNum |
| vector< int > | m\_HabitatTable\_Size |
| vector< double > | m\_InsectTable |
| Skylark\_Female \* | MyMate |
| Public Attributes inherited from Skylark\_Adult | |
| bool | Paired |
| Public Attributes inherited from Skylark\_Base | |
| int | Age |
| int | m\_Born\_x |
| int | m\_Born\_y |
| TTypesOfSkState | m\_CurrentSkState |
| int | m\_MyHome |
|  | The vegetation type where the skylark was born. |
| Skylark\_Population\_Manager \* | m\_OurPopulationManager |
| SkTerritories \* | m\_OurTerritories |
| double | m\_pcide\_conc |
| double | m\_pesticide\_accumulation |
| double | m\_Size |

|  |  |
| --- | --- |
| Protected Member Functions | |
| void | ConstructAHabitatTable () |
| int | EstablishingATerritory () |
| double | GetFood (int time) |
| int | GetMigrationMortality () |
| virtual bool | OnFarmEvent (FarmToDo event) |
| void | OptimiseHabitatSearchingOrder () |
| virtual void | PesticideResponse () |
| void | ReEvaluateTerritory () |
|  | Daily re-evaluation of territory. |
| bool | st\_Arriving () |
| int | st\_AttractingAMate () |
| int | st\_CaringForYoung () |
| void | st\_Dying () |
| int | st\_Emigrating () |
| int | st\_FindingTerritory () |
| TTypesOfSkState | st\_Floating () |
| int | st\_Flocking () |
| int | st\_FollowingMate () |
| bool | st\_Immigrating () |
| int | st\_ScaringOffChicks () |
| int | st\_TempLeavingArea () |
| Protected Member Functions inherited from Skylark\_Adult | |
| bool | GetBadWeather () |
|  | Extreme weather conditions check. |
| double | GetVegHindrance (int PolyRef) |
| double | GetWeatherHindrance () |
| virtual double | RemoveEM (double food) |
| Protected Member Functions inherited from Skylark\_Base | |
| bool | DailyMortality (int mort) |
| Protected Member Functions inherited from TAnimal | |
| void | CorrectWrapRound (void) |
|  | Corrects wrap around co-ordinate problems. |

|  |  |
| --- | --- |
| Protected Attributes | |
| int | GoodWeather |
| Skylark\_Nestling \* | m\_Brood [26] |
| int | m\_BroodSize |
| bool | m\_firstPF |
| int | m\_MyMinTerritoryQual |
| bool | m\_pesticide\_sprayed |
| double | m\_XFNestAcceptScore |
| int | No\_HabitatTable\_Refs |
| Protected Attributes inherited from Skylark\_Adult | |
| bool | BSuccess |
| vector< Point > \* | m\_aTerrlist |
| double | MyExtractEff |
| skTerritory\_struct | MyTerritory |

---

## Constructor & Destructor Documentation

|  |  |  |  |
| --- | --- | --- | --- |
| Skylark\_Male::Skylark\_Male | ( | int | *x*, |
|  |  | int | *y*, |
|  |  | double | *size*, |
|  |  | int | *age*, |
|  |  | SkTerritories \* | *Terrs*, |
|  |  | Landscape \* | *L*, |
|  |  | Skylark\_Population\_Manager \* | *SPM*, |
|  |  | int | *bx*, |
|  |  | int | *by*, |
|  |  | int | *mh* |
|  | ) |  |  |

The Male constructor. This initialises some important parameters.

There is some individual variation around the territory acceptance score, up to 10% max

References Skylark\_Adult::BSuccess, g\_rand\_uni, GoodWeather, HaveTerritory, m\_Brood, m\_BroodSize, m\_firstPF, m\_MyMinTerritoryQual, m\_XFNestAcceptScore, MyMate, and CfgFloat::value().

: Skylark\_Adult( x, y, size, age, Terrs, L, SPM, bx, by, mh ) {

GoodWeather = 0;

HaveTerritory = false;

MyMate = NULL;

m\_BroodSize = 0; // don't start life with chicks!

BSuccess = false;

m\_firstPF = false;

m\_MyMinTerritoryQual = (int) (cfg\_FemaleMinTerritoryAcceptScore.value() \* (g\_rand\_uni()/10.0 + 1.0)); //= 1.0 to 1.1

m\_XFNestAcceptScore = m\_MyMinTerritoryQual \* (double) (g\_rand\_uni()+2.1);

for ( int i = 0; i < 6; i++ ) m\_Brood[ i ] = NULL;

}

|  |  |  |  |  |
| --- | --- | --- | --- | --- |
| Skylark\_Male::~Skylark\_Male | ( |  | ) |  |

{

}

---

## Member Function Documentation

|  |  |  |  |  |  |
| --- | --- | --- | --- | --- | --- |
| void Skylark\_Male::BeginStep | ( | void |  | ) |  |

virtual

Reimplemented from TAnimal.

References TAnimal::CheckManagement(), Skylark\_Base::m\_CurrentSkState, ReEvaluateTerritory(), st\_CaringForYoung(), st\_Floating(), TALMaSSObject::StepDone, toss\_AttractingAMate, toss\_Destroy, toss\_FollowingMate, toss\_MCaringForYoung, toss\_MFloating, and toss\_ScaringOffChicks.

{

#ifdef \_\_CJTDebug\_5

if ( IsAlive() != 0x0DEADC0DE ) DEADCODEError();

#endif

if ( m\_CurrentSkState == toss\_Destroy ) return;

CheckManagement();

switch ( m\_CurrentSkState ) {

case toss\_MFloating: // Floating

m\_CurrentSkState=st\_Floating(); // Legal returns are toss\_MFlocking, toss\_MFindingTerritory, toss\_MTempLeavingArea

StepDone = true;

break;

case toss\_MCaringForYoung: // CaringForYoung

if ( st\_CaringForYoung() )

m\_CurrentSkState = toss\_ScaringOffChicks; //ScareOffChicks

StepDone = true;

case toss\_AttractingAMate:

case toss\_ScaringOffChicks:

case toss\_FollowingMate:

ReEvaluateTerritory();

break;

default:

/\* char errornum[20]; sprintf(errornum, "%d", m\_CurrentSkState );

g\_land->Warn("Skylark\_Male::BeginStep(): Unknown state: ", errornum); exit(0); \*/

break;

}

}

|  |  |  |  |  |
| --- | --- | --- | --- | --- |
| void Skylark\_Male::ConstructAHabitatTable | ( |  | ) |  |

protected

References m\_HabitatTable\_PNum, m\_HabitatTable\_Size, skTTerritory::m\_hr\_polys, skTTerritory::m\_hr\_sizes, m\_InsectTable, Skylark\_Base::m\_OurTerritories, Skylark\_Adult::MyTerritory, No\_HabitatTable\_Refs, skTerritory\_struct::ref, and SkTerritories::Supply\_terr().

Referenced by OnPairing().

{

#ifdef \_\_CJTDebug\_5

if ( IsAlive() != 0xDEADC0DE )

DEADCODEError();

#endif

// This function must be called when the female arrives and

// again when the eggs first hatch

// This function constructs a table listing

// the reference of each habitat object

// and the area of that object within the skylarks home range

// First must find out which habitats are in HomeRange

// this is done by searching the landscape raster and counting how many

// 1 meter areas are present for each habitat object

// These days this is easy, as the information is already

// available in the territory as a pre-chewed list provided

// for this very use by PreProcessLandscape2()! :-)

skTTerritory \* l\_terr = m\_OurTerritories->Supply\_terr( MyTerritory.ref );

int l\_polys = (int)l\_terr->m\_hr\_polys.size();

#ifdef \_\_PESTICIDE\_RA

m\_PConcTable.resize(l\_polys);

#endif

m\_HabitatTable\_PNum.resize(l\_polys);

m\_HabitatTable\_Size.resize(l\_polys);

m\_InsectTable.resize(l\_polys);

for ( int i = 0; i < l\_polys; i++ ) {

m\_HabitatTable\_PNum[ i ] = l\_terr->m\_hr\_polys[ i ];

m\_HabitatTable\_Size[ i ] = l\_terr->m\_hr\_sizes[ i ];

}

No\_HabitatTable\_Refs = l\_polys;

}

|  |  |  |  |  |
| --- | --- | --- | --- | --- |
| int Skylark\_Male::DefendTerritory | ( |  | ) |  |

References Skylark\_Base::Age, Skylark\_Adult::BSuccess, and Skylark\_Adult::Paired.

Referenced by st\_FindingTerritory().

{

#ifdef \_\_CJTDebug\_5

if ( IsAlive() != 0xDEADC0DE )

DEADCODEError();

#endif

if ( ( Paired == true ) || ( BSuccess == true ) ) return 999; // very old

else

return Age; //can't be ousted if paired or owned the territory before

}

|  |  |  |  |  |  |
| --- | --- | --- | --- | --- | --- |
| void Skylark\_Male::EndStep | ( | void |  | ) |  |

virtual

Reimplemented from TAnimal.

References Skylark\_Base::m\_CurrentSkState, PesticideResponse(), and toss\_Destroy.

{

#ifdef \_\_CJTDebug\_5

if ( IsAlive() != 0x0DEADC0DE ) DEADCODEError();

#endif

if ( m\_CurrentSkState == toss\_Destroy ) return;

#ifdef \_\_PESTICIDE\_RA

PesticideResponse();

#endif

}

|  |  |  |  |  |
| --- | --- | --- | --- | --- |
| int Skylark\_Male::EstablishingATerritory | ( |  | ) |  |

protected

References HaveTerritory, Skylark\_Adult::m\_aTerrlist, TAnimal::m\_Location\_x, TAnimal::m\_Location\_y, Skylark\_Base::m\_OurPopulationManager, Skylark\_Base::m\_OurTerritories, Skylark\_Adult::MyTerritory, SkTerritories::Occupy(), skTerritory\_struct::ref, Population\_Manager::SimH, Population\_Manager::SimW, skTerritory\_struct::size, skTerritory\_struct::x, and skTerritory\_struct::y.

Referenced by OnReHouse(), and Step().

{

/\*

The male claims the territory, primarily a programming construct, but resulting in the male being registered as occupying the territory.<br>

\*/

#ifdef \_\_CJTDebug\_5

if ( IsAlive() != 0x0DEADC0DE ) DEADCODEError();

#endif

m\_aTerrlist->clear();

// Stand in the centre.

m\_Location\_x = MyTerritory.x + ( MyTerritory.size / 2 );

if ( m\_Location\_x >= m\_OurPopulationManager->SimW ) m\_Location\_x = m\_Location\_x % m\_OurPopulationManager->SimW;

m\_Location\_y = MyTerritory.y + ( MyTerritory.size / 2 );

if ( m\_Location\_y >= m\_OurPopulationManager->SimH ) m\_Location\_y = m\_Location\_y % m\_OurPopulationManager->SimH;

// claim occupation

m\_OurTerritories->Occupy( MyTerritory.ref, this );

HaveTerritory = true;

return 0; // TransferToState(AttractMate)

}

|  |  |  |  |  |  |
| --- | --- | --- | --- | --- | --- |
| double Skylark\_Male::GetFood | ( | int | *time* | ) |  |

protected

References Skylark\_Adult::GetVegHindrance(), Skylark\_Adult::GetWeatherHindrance(), KcalPerGInsect\_kg\_inv, m\_HabitatTable\_PNum, m\_HabitatTable\_Size, m\_InsectTable, TAnimal::m\_OurLandscape, Skylark\_Adult::MyExtractEff, No\_HabitatTable\_Refs, TALMaSSObject::OnArrayBoundsError(), OptimiseHabitatSearchingOrder(), Landscape::SupplyInsects(), and Landscape::SupplyPesticide().

Referenced by st\_CaringForYoung(), and st\_FollowingMate().

{

#ifdef \_\_CJTDebug\_5

if ( IsAlive() != 0x0DEADC0DE ) DEADCODEError();

#endif

#ifdef \_\_CJTDebug\_4

if ( No\_HabitatTable\_Refs > 250 )

OnArrayBoundsError();

#endif

// This must be called each day before the female's code is executed

double food = 0;

// First find out how much food is available from each habitat

// Note that the pesticide risk assessment code is integrated here.

// Stage 1 create the insect data

for ( int i = 0; i < No\_HabitatTable\_Refs; i++ ) {

// Insects = Insects \* Hindrance

// Insect table holds insects per squaremetre

#ifdef \_\_PESTICIDE\_RA

m\_PConcTable[i] = m\_OurLandscape->SupplyPesticide( m\_HabitatTable\_PNum[ i ] );

#endif

double WHindrance = GetWeatherHindrance();

double hind = GetVegHindrance( m\_HabitatTable\_PNum[ i ] );

double insects = m\_OurLandscape->SupplyInsects( m\_HabitatTable\_PNum[ i ] );

// Insects = Insects \* Hindrance

// Insect table holds insects per squaremetre

m\_InsectTable[ i ] = insects \* hind \* WHindrance;

}

// Now sort habitats into optimal searching order (i.e. most insect/m2)

OptimiseHabitatSearchingOrder();

// Now extract insects available from each for as long as spends searching

int index = 0;

// Note at present this routine assumes that 1 min is spent per m2

// if this assumption is changed then we must alter 'time' accordingly

#ifdef \_\_PESTICIDE\_RA

m\_pcide = 0.0;

#endif

double fd;

while ( time > 0 ) {

int area = m\_HabitatTable\_Size[ index ];

if ( time > area) {

time -= area;

// Food+= area\*WeatherHindrance\*ExtractionEfficiency\*Insects per m2

fd = area \* MyExtractEff \* m\_InsectTable[ index ];

food += fd;

#ifdef \_\_PESTICIDE\_RA

m\_pcide += fd \* m\_PConcTable[ index ] \* KcalPerGInsect\_kg\_inv; // Needs converting from kcal to mg;

#endif

index++;

#ifdef \_\_CJTDebug\_4

if ( index > 249 )

OnArrayBoundsError();

#endif

} else {

// Food+=

// minutes left\*WeatherHindrance\*ExtractionEfficiency\*Insects per m2

fd = time \* MyExtractEff \* m\_InsectTable[ index ];

food += fd;

#ifdef \_\_PESTICIDE\_RA

m\_pcide += m\_PConcTable[ index ] \* time \* KcalPerGInsect\_kg\_inv; // Needs converting from kcal to mg;

#endif

time = 0;

}

}

return food;

}

|  |  |  |  |  |
| --- | --- | --- | --- | --- |
| int Skylark\_Male::GetMigrationMortality | ( |  | ) |  |

protected

References Skylark\_Base::Age, JuvenileReturnMort, Skylark\_Base::m\_OurPopulationManager, and Skylark\_Population\_Manager::SupplyM\_Mig\_Mort().

Referenced by st\_Immigrating().

{

#ifdef \_\_CJTDebug\_5

if ( IsAlive() != 0x0DEADC0DE ) DEADCODEError();

#endif

Age++;

if ( Age == 1 ) return JuvenileReturnMort;

return m\_OurPopulationManager->SupplyM\_Mig\_Mort();

}

|  |  |  |  |  |  |
| --- | --- | --- | --- | --- | --- |
| void Skylark\_Male::OnAddNestling | ( | Skylark\_Nestling \* | *N* | ) |  |

References m\_Brood, and TALMaSSObject::OnArrayBoundsError().

Referenced by Skylark\_Population\_Manager::CreateObjects().

{

#ifdef \_\_CJTDebug\_5

if ( IsAlive() != 0xDEADC0DE )

DEADCODEError();

#endif

// Add nestling and record the new brood member

m\_Brood[ m\_BroodSize++ ] = N;

#ifdef \_\_CJTDebug\_4

if ( m\_BroodSize > 25 ) {

OnArrayBoundsError();

}

#endif

}

|  |  |  |  |
| --- | --- | --- | --- |
| void Skylark\_Male::OnAddPreFledgeling | ( | Skylark\_PreFledgeling \* | *P*, |
|  |  | Skylark\_Nestling \* | *N* |
|  | ) |  |  |

References m\_Brood, m\_BroodSize, m\_firstPF, MyMate, Skylark\_Female::OnBreedSuccess(), and Landscape::Warn().

Referenced by Skylark\_Population\_Manager::CreateObjects().

{

#ifdef \_\_CJTDebug\_5

if ( IsAlive() != 0xDEADC0DE )

DEADCODEError();

#endif

// Must now replace nestling pointer with one to the new object

int n = -1;

for ( int i = 0; i < m\_BroodSize; i++ ) {

if ( N == m\_Brood[ i ] ) n = i;

}

if ( n == -1 ) {

// Very nasty problems

g\_land->Warn( "Skylark\_Male::Step: Maturing PreFl. not in Male's list", NULL );

exit( 1 );

}

m\_Brood[ n ] = P;

if ( !m\_firstPF ) {

MyMate->OnBreedSuccess();

}

m\_firstPF = true;

}

|  |  |  |  |  |
| --- | --- | --- | --- | --- |
| void Skylark\_Male::OnBroodDeath | ( |  | ) |  |

References HaveTerritory, m\_Brood, m\_BroodSize, Skylark\_Base::m\_CurrentSkState, MyMate, Skylark\_Female::OnBroodDeath(), Skylark\_Adult::Paired, toss\_AttractingAMate, toss\_FollowingMate, and toss\_MFlocking.

Referenced by OnBroodDesertion(), OnNestlingDeath(), OnNestPredatation(), and OnPreFledgelingDeath().

{

#ifdef \_\_CJTDebug\_5

if ( IsAlive() != 0xDEADC0DE )

DEADCODEError();

#endif

for ( int i = 0; i < m\_BroodSize; i++ ) m\_Brood[ i ] = NULL;

m\_BroodSize = 0;

if ( Paired ) {

m\_CurrentSkState = toss\_FollowingMate; // Follow mate

MyMate->OnBroodDeath();

} else if ( HaveTerritory ) m\_CurrentSkState = toss\_AttractingAMate;

else {

m\_CurrentSkState = toss\_MFlocking;

}

}

|  |  |  |  |  |
| --- | --- | --- | --- | --- |
| void Skylark\_Male::OnBroodDesertion | ( |  | ) |  |

References m\_Brood, m\_BroodSize, OnBroodDeath(), and Skylark\_Nestling::OnDeserted().

{

for ( int i = 0; i < m\_BroodSize; i++ ) {

m\_Brood[ i ]->OnDeserted();

}

m\_BroodSize = 0;

OnBroodDeath();

}

|  |  |  |  |  |
| --- | --- | --- | --- | --- |
| void Skylark\_Male::OnEggHatch | ( |  | ) |  |

References Skylark\_Base::m\_CurrentSkState, toss\_MCaringForYoung, and Landscape::Warn().

Referenced by Skylark\_Female::OnEggsHatch().

{

#ifdef \_\_CJTDebug\_5

if ( IsAlive() != 0xDEADC0DE )

DEADCODEError();

#endif

// NB this must not occur if there are still chicks to scare off -

// if it does then they are left in limbo!

// Brood information is already put here by CreatObjects

#ifdef \_\_CJTDebug\_7

if ( m\_BroodSize < 1 ) {

g\_land->Warn( "Skylark\_Male::OnEggHatch: DeBug7 ", NULL ); exit( 1 );

}

#endif

m\_CurrentSkState = toss\_MCaringForYoung; // TransferToState(CareForYoung);

}

|  |  |  |  |  |
| --- | --- | --- | --- | --- |
| bool Skylark\_Male::OnEvicted | ( |  | ) |  |

References SkTerritories::GetTerritoriesByDistance(), HaveTerritory, Skylark\_Adult::m\_aTerrlist, Skylark\_Base::m\_CurrentSkState, Skylark\_Base::m\_OurPopulationManager, Skylark\_Base::m\_OurTerritories, Skylark\_Adult::MyTerritory, Skylark\_Adult::Paired, skTerritory\_struct::ref, SkTerritories::RemoveMale(), Skylark\_Population\_Manager::TheSkylarkTerrs, toss\_MFindingTerritory, skTerritory\_struct::x, and skTerritory\_struct::y.

Referenced by st\_FindingTerritory().

{

#ifdef \_\_CJTDebug\_5

if ( IsAlive() != 0xDEADC0DE )

DEADCODEError();

#endif

// returns true if evicted or otherwise false

if ( !Paired ) {

m\_aTerrlist->clear();

m\_OurPopulationManager->TheSkylarkTerrs->GetTerritoriesByDistance(MyTerritory.x, MyTerritory.y, m\_aTerrlist);

m\_OurTerritories->RemoveMale( MyTerritory.ref );

MyTerritory.ref = -1;

HaveTerritory = false;

m\_CurrentSkState = toss\_MFindingTerritory;

return true;

} else return false; // can't evict I'm paired

}

|  |  |  |  |  |  |
| --- | --- | --- | --- | --- | --- |
| bool Skylark\_Male::OnFarmEvent | ( | FarmToDo | *event* | ) |  |

protectedvirtual

Reimplemented from TAnimal.

References autumn\_harrow, autumn\_or\_spring\_plough, autumn\_plough, autumn\_roll, autumn\_sow, burn\_straw\_stubble, cattle\_out, cattle\_out\_low, cut\_to\_hay, cut\_to\_silage, cut\_weeds, Calendar::Date(), deep\_ploughing, Landscape::EventtypeToString(), fa\_ammoniumsulphate, fa\_greenmanure, fa\_manure, fa\_npk, fa\_pk, fa\_sludge, fa\_slurry, fp\_greenmanure, fp\_liquidNH3, fp\_manganesesulphate, fp\_manure, fp\_npk, fp\_npks, fp\_pk, fp\_sludge, fp\_slurry, fungicide\_treat, g\_date, glyphosate, growth\_regulator, harvest, hay\_bailing, hay\_turning, herbicide\_treat, hilling\_up, insecticide\_treat, Skylark\_Base::m\_CurrentSkState, TAnimal::m\_OurLandscape, molluscicide, mow, pigs\_out, product\_treat, random(), row\_cultivation, sleep\_all\_day, spring\_harrow, spring\_plough, spring\_roll, spring\_sow, straw\_chopping, strigling, strigling\_sow, stubble\_harrowing, swathing, syninsecticide\_treat, toss\_MDying, trial\_insecticidetreat, CfgInt::value(), Landscape::Warn(), water, and winter\_plough.

{

switch ( event ) {

case sleep\_all\_day:

break;

case autumn\_plough:

break;

case autumn\_harrow:

break;

case autumn\_roll:

break;

case autumn\_sow:

break;

case winter\_plough:

break;

case deep\_ploughing:

break;

case spring\_plough:

break;

case spring\_harrow:

break;

case spring\_roll:

break;

case spring\_sow:

break;

case fp\_npks:

break;

case fp\_npk:

break;

case fp\_pk:

break;

case fp\_liquidNH3:

break;

case fp\_slurry:

break;

case fp\_manganesesulphate:

break;

case fp\_manure:

break;

case fp\_greenmanure:

break;

case fp\_sludge:

break;

case fa\_npk:

break;

case fa\_pk:

break;

case fa\_slurry:

break;

case fa\_ammoniumsulphate:

break;

case fa\_manure:

break;

case fa\_greenmanure:

break;

case fa\_sludge:

break;

case herbicide\_treat:

break;

case growth\_regulator:

break;

case fungicide\_treat:

break;

case insecticide\_treat:

case trial\_insecticidetreat:

break;

case syninsecticide\_treat:

if ( random( 1000 ) < cfg\_insecticide\_direct\_mortM.value() ) {

#ifdef TEST\_ISSUE\_DEATH\_WARRANT

printf( "Skylark\_Male::OnFarmEvent() : %d : %d\n", event, ( int )g\_date->Date() );

#endif

m\_CurrentSkState = toss\_MDying;

}

break;

case molluscicide:

break;

case row\_cultivation:

break;

case strigling:

break;

case hilling\_up:

break;

case water:

break;

case swathing:

break;

case harvest:

break;

case cattle\_out:

case cattle\_out\_low:

break;

case cut\_to\_hay:

break;

case cut\_to\_silage:

break;

case straw\_chopping:

break;

case hay\_turning:

break;

case hay\_bailing:

break;

case stubble\_harrowing:

break;

case autumn\_or\_spring\_plough:

break;

case burn\_straw\_stubble:

break;

case mow:

break;

case cut\_weeds:

break;

case pigs\_out:

break;

case strigling\_sow:

break;

case product\_treat:

break;

case glyphosate:

break;

default:

g\_land->Warn( "Skylark\_Male::OnFarmEvent(): ""Unknown event type:", m\_OurLandscape->EventtypeToString( event ) );

exit( 1 );

}

if ( m\_CurrentSkState == toss\_MDying ) return true; else

return false;

}

|  |  |  |  |
| --- | --- | --- | --- |
| double Skylark\_Male::OnFoodMessage | ( | int | *n*, |
|  |  | double | *f*, |
|  |  | double | *p* |
|  | ) |  |  |

inline

References UNREFERENCED\_PARAMETER.

Referenced by Skylark\_Female::FeedYoung().

{

#ifndef \_\_PESTICIDE\_RA\_CHICK

UNREFERENCED\_PARAMETER(p);

#endif

double extra = m\_Brood[n]->On\_FoodSupply( f );

#ifdef \_\_PESTICIDE\_RA\_CHICK

m\_Brood[n]->m\_pesticide\_accumulation+= (((f-extra)/f)\*p);

#endif

return extra;

}

|  |  |  |  |  |
| --- | --- | --- | --- | --- |
| void Skylark\_Male::OnMateDying | ( |  | ) |  |

References Skylark\_Adult::BSuccess, g\_stopdate, m\_Brood, m\_BroodSize, Skylark\_Base::m\_CurrentSkState, TAnimal::m\_OurLandscape, MyMate, Skylark\_Nestling::OnDadDead(), Skylark\_Adult::Paired, Landscape::SupplyDayInYear(), toss\_AttractingAMate, and toss\_MFlocking.

Referenced by Skylark\_Population\_Manager::Catastrophe(), and Skylark\_Female::st\_Dying().

{

#ifdef \_\_CJTDebug\_5

if ( IsAlive() != 0xDEADC0DE )

DEADCODEError();

#endif

// Brood size is reduced to zero because he forgets about the young

// So must also tell them to forget him

for ( int i = 0; i < m\_BroodSize; i++ ) {

m\_Brood[ i ]->OnDadDead();

m\_Brood[ i ] = NULL;

}

m\_BroodSize = 0;

MyMate = NULL;

Paired = false;

BSuccess = false;

int today = m\_OurLandscape->SupplyDayInYear();

if ( today >= g\_stopdate ) {

m\_CurrentSkState = toss\_MFlocking;

}

else m\_CurrentSkState = toss\_AttractingAMate; // TransferToState(AttractAMate);

}

|  |  |  |  |  |
| --- | --- | --- | --- | --- |
| void Skylark\_Male::OnMateLeaving | ( |  | ) |  |

References Skylark\_Adult::BSuccess, g\_stopdate, skTTerritory::GetQuality(), HaveTerritory, m\_Brood, m\_BroodSize, Skylark\_Base::m\_CurrentSkState, m\_MyMinTerritoryQual, TAnimal::m\_OurLandscape, Skylark\_Base::m\_OurTerritories, MyMate, Skylark\_Adult::MyTerritory, skTerritory\_struct::nqual, Skylark\_Nestling::OnDadDead(), Skylark\_Adult::Paired, skTerritory\_struct::ref, SkTerritories::RemoveMale(), SkTerritories::Supply\_terr(), Landscape::SupplyDayInYear(), toss\_AttractingAMate, and toss\_MFlocking.

Referenced by Skylark\_Female::st\_GivingUpTerritory(), and Skylark\_Female::st\_StoppingBreeding().

{

#ifdef \_\_CJTDebug\_5

if ( IsAlive() != 0xDEADC0DE )

DEADCODEError();

#endif

// Brood size is reduced to zero because he forgets about the young

// He must tell them to forget about him as well

for ( int i = 0; i < m\_BroodSize; i++ ) {

m\_Brood[ i ]->OnDadDead(); // OK he is lying

m\_Brood[ i ] = NULL;

}

m\_BroodSize = 0;

if ( !BSuccess ) {

// She has left him, so in this case he gives up

MyMate = NULL;

double qual = m\_OurTerritories->Supply\_terr( MyTerritory.ref )->GetQuality();

// Store the quality for today so the female can read it

if (( m\_OurLandscape->SupplyDayInYear() < g\_stopdate ) && (qual > m\_MyMinTerritoryQual))

{

MyTerritory.nqual = qual;

m\_CurrentSkState = toss\_AttractingAMate; // TransferToState(AttractAMate);

}

else

{

// Too late to try again

m\_CurrentSkState = toss\_MFlocking;

HaveTerritory = false;

// Must de-register himself as territory owner

m\_OurTerritories->RemoveMale( MyTerritory.ref );

// Just to be sure we can set the ref to -1 = no territory

MyTerritory.ref = -1;

}

} else {

// She has stopped breeding for the year

m\_CurrentSkState = toss\_MFlocking; // TransferToState(Flocking);

HaveTerritory = false;

// Must de-register himself as territory owner

m\_OurTerritories->RemoveMale( MyTerritory.ref );

// Just to be sure we can set the ref to -1 = no territory

MyTerritory.ref = -1;

}

// In all cases

Paired = false;

}

|  |  |  |  |  |  |
| --- | --- | --- | --- | --- | --- |
| void Skylark\_Male::OnMateNeverComesBack | ( | Skylark\_Female \* | *AFemale* | ) |  |

References Skylark\_Adult::BSuccess, MyMate, and Skylark\_Adult::Paired.

Referenced by Skylark\_Female::st\_Finding\_Territory(), and Skylark\_Female::st\_Immigrating().

{

#ifdef \_\_CJTDebug\_5

if ( IsAlive() != 0xDEADC0DE )

DEADCODEError();

#endif

// Just a safety check

if ( MyMate == AFemale ) {

MyMate = NULL;

Paired = false;

BSuccess = false;

}

}

|  |  |  |  |  |  |
| --- | --- | --- | --- | --- | --- |
| void Skylark\_Male::OnNestlingDeath | ( | Skylark\_Nestling \* | *N* | ) |  |

References m\_Brood, m\_BroodSize, TALMaSSObject::OnArrayBoundsError(), OnBroodDeath(), and Landscape::Warn().

Referenced by Skylark\_Nestling::st\_Dying().

{

#ifdef \_\_CJTDebug\_5

if ( IsAlive() != 0xDEADC0DE )

DEADCODEError();

bool found = false;

#endif

// remove the dead one

m\_BroodSize--;

#ifdef \_\_CJTDebug\_4

if ( m\_BroodSize < 0 ) {

OnArrayBoundsError();

}

#endif

for ( int i = 0; i <= m\_BroodSize; i++ ) {

if ( m\_Brood[ i ] == N ) {

m\_Brood[ i ] = m\_Brood[ m\_BroodSize ];

m\_Brood[ m\_BroodSize ] = NULL;

#ifdef \_\_CJTDebug\_5

found = true;

#endif

}

}

#ifdef \_\_CJTDebug\_5

if ( found == false ) {

g\_land->Warn( "Skylark\_Male::OnNestlingDeath - Debug 5 ", NULL ); exit( 1 );

}

#endif

// If all dead do on Brood death

// remove the matured one

if ( m\_BroodSize == 0 ) {

OnBroodDeath();

}

}

|  |  |  |  |
| --- | --- | --- | --- |
| void Skylark\_Male::OnNestLocation | ( | int | *x*, |
|  |  | int | *y* |
|  | ) |  |  |

References TAnimal::m\_Location\_x, and TAnimal::m\_Location\_y.

Referenced by Skylark\_Female::st\_MakingNest().

{

#ifdef \_\_CJTDebug\_5

if ( IsAlive() != 0xDEADC0DE )

DEADCODEError();

#endif

if ( x == 0 ) x = 2;

m\_Location\_x = x - 1;

m\_Location\_y = y;

}

|  |  |  |  |  |
| --- | --- | --- | --- | --- |
| void Skylark\_Male::OnNestPredatation | ( |  | ) |  |

References m\_Brood, m\_BroodSize, OnBroodDeath(), and Skylark\_Nestling::OnYouHaveBeenEaten().

Referenced by Skylark\_Nestling::EndStep().

{

for ( int i = 0; i < m\_BroodSize; i++ ) {

m\_Brood[ i ]->OnYouHaveBeenEaten();

}

m\_BroodSize = 0;

OnBroodDeath();

}

|  |  |  |  |  |  |
| --- | --- | --- | --- | --- | --- |
| void Skylark\_Male::OnPairing | ( | Skylark\_Female \* | *female* | ) |  |

References Skylark\_Adult::BSuccess, ConstructAHabitatTable(), Skylark\_Base::m\_CurrentSkState, MyMate, Skylark\_Female::OnMaleNeverComesBack(), Skylark\_Adult::Paired, and toss\_FollowingMate.

Referenced by Skylark\_Female::EstablishTerritory().

{

#ifdef \_\_CJTDebug\_5

if ( IsAlive() != 0xDEADC0DE )

DEADCODEError();

#endif

Paired = true;

if ( MyMate ) {

if ( MyMate != female ) MyMate->OnMaleNeverComesBack( this );

}

MyMate = female;

ConstructAHabitatTable();

BSuccess = false;

m\_CurrentSkState = toss\_FollowingMate;

}

|  |  |  |  |  |  |
| --- | --- | --- | --- | --- | --- |
| void Skylark\_Male::OnPreFledgelingDeath | ( | Skylark\_PreFledgeling \* | *P* | ) |  |

References m\_Brood, m\_BroodSize, TALMaSSObject::OnArrayBoundsError(), OnBroodDeath(), and Landscape::Warn().

Referenced by Skylark\_PreFledgeling::st\_Dying().

{

#ifdef \_\_CJTDebug\_5

if ( IsAlive() != 0xDEADC0DE )

DEADCODEError();

bool found = false;

#endif

// remove the dead one

for ( int i = 0; i < m\_BroodSize; i++ ) {

if ( m\_Brood[ i ] == P ) {

m\_Brood[ i ] = m\_Brood[ --m\_BroodSize ];

m\_Brood[ m\_BroodSize ] = NULL;

#ifdef \_\_CJTDebug\_5

found = true;

#endif

}

}

#ifdef \_\_CJTDebug\_4

if ( m\_BroodSize < 0 ) {

OnArrayBoundsError();

}

#endif

#ifdef \_\_CJTDebug\_5

if ( found == false ) {

g\_land->Warn( "Skylark\_Male::OnPreFledgelingDeath: DeBug5 ", NULL );

exit( 1 );

}

#endif

// If all dead do on Brood death

// remove the matured one

if ( m\_BroodSize == 0 ) {

OnBroodDeath();

}

}

|  |  |  |  |  |  |
| --- | --- | --- | --- | --- | --- |
| void Skylark\_Male::OnPreFledgelingMature | ( | Skylark\_PreFledgeling \* | *P* | ) |  |

References Skylark\_Adult::BSuccess, HaveTerritory, m\_Brood, m\_BroodSize, Skylark\_Base::m\_CurrentSkState, MyMate, TALMaSSObject::OnArrayBoundsError(), Skylark\_Female::OnBreedingSuccess(), Skylark\_Female::OnStopFeedingChicks(), Skylark\_Adult::Paired, toss\_AttractingAMate, toss\_FollowingMate, and toss\_MFlocking.

Referenced by Skylark\_PreFledgeling::st\_Maturing().

{

#ifdef \_\_CJTDebug\_5

if ( IsAlive() != 0xDEADC0DE )

DEADCODEError();

#endif

for ( int i = 0; i < m\_BroodSize; i++ ) {

if ( m\_Brood[ i ] == P ) {

m\_Brood[ i ] = m\_Brood[ --m\_BroodSize ];

m\_Brood[ m\_BroodSize ] = NULL;

BSuccess = true;

if ( MyMate ) MyMate->OnBreedingSuccess();

}

}

// If all matured do on Brood death

// remove the matured one

#ifdef \_\_CJTDebug\_4

if ( m\_BroodSize < 0 ) {

OnArrayBoundsError();

}

#endif

if ( m\_BroodSize == 0 ) {

if ( Paired ) {

// it all depends what the female is doing

// what he must do.

// if she is also feeding he needs to tell here to stop

MyMate->OnStopFeedingChicks();

m\_CurrentSkState = toss\_FollowingMate;

} else if ( HaveTerritory ) m\_CurrentSkState = toss\_AttractingAMate;

else {

m\_CurrentSkState = toss\_MFlocking;

}

}

}

|  |  |  |  |  |
| --- | --- | --- | --- | --- |
| void Skylark\_Male::OnReHouse | ( |  | ) |  |

References EstablishingATerritory(), Skylark\_Female::EstablishTerritory(), SkTerritories::FemaleOccupy(), HaveTerritory, Skylark\_Base::m\_CurrentSkState, Skylark\_Base::m\_OurTerritories, MyMate, Skylark\_Adult::MyTerritory, Skylark\_Female::OnMateHomeless(), Skylark\_Adult::Paired, skTerritory\_struct::ref, skTerritory\_struct::size, SkTerritories::Supply\_Owner(), SkTerritories::Supply\_size(), SkTerritories::Supply\_x(), SkTerritories::Supply\_y(), SkTerritories::SupplyIsNestValid(), SkTerritories::SupplyNoTerritories(), toss\_MFindingTerritory, skTerritory\_struct::validnest, skTerritory\_struct::x, and skTerritory\_struct::y.

Referenced by Skylark\_Population\_Manager::ReHouse().

{

#ifdef \_\_CJTDebug\_5

if ( IsAlive() != 0xDEADC0DE )

DEADCODEError();

#endif

int mindist=100000000;

int index=-1;

// Needs to sort through the new list of territories to find one closest to its m\_Location\_x, m\_Location\_y

int Nterrs = m\_OurTerritories->SupplyNoTerritories();

int dist=0;

for (int i=0; i<Nterrs; i++) {

Skylark\_Base\* TheOwner = m\_OurTerritories->Supply\_Owner( i );

if ( !TheOwner ) {

int tx = m\_OurTerritories->Supply\_x(i);

int ty = m\_OurTerritories->Supply\_y(i);

dist = (int)sqrt((float)(tx\*tx+ty\*ty));

if (dist< mindist) {

mindist=dist;

index=i;

}

}

}

if (index!=-1) {

MyTerritory.ref = index;

// store territory data

MyTerritory.x = m\_OurTerritories->Supply\_x( index );

MyTerritory.y = m\_OurTerritories->Supply\_y( index );

MyTerritory.size = m\_OurTerritories->Supply\_size( index );

MyTerritory.validnest = m\_OurTerritories->SupplyIsNestValid(index);

EstablishingATerritory();

if (Paired) {

m\_OurTerritories->FemaleOccupy( index, MyMate );

MyMate->EstablishTerritory();

}

}

else {

if (Paired) {

MyMate->OnMateHomeless();

MyMate = NULL;

Paired=false;

}

MyTerritory.ref = -1;

HaveTerritory = false;

m\_CurrentSkState = toss\_MFindingTerritory;

}

}

|  |  |  |  |  |
| --- | --- | --- | --- | --- |
| void Skylark\_Male::OptimiseHabitatSearchingOrder | ( |  | ) |  |

protected

References m\_HabitatTable\_PNum, m\_HabitatTable\_Size, m\_InsectTable, and No\_HabitatTable\_Refs.

Referenced by GetFood().

{

#ifdef \_\_CJTDebug\_5

if ( IsAlive() != 0xDEADC0DE )

DEADCODEError();

#endif

// This method sorts the HabitatTable on the basis of the highest

// available insects per square metre basis

// It also puts the available insects into the table

// Sort the entries on the basis of InsectTable

int hold1;

double hold2;

int interChange = 1;

int pass = 0;

while ( ( pass++ <= No\_HabitatTable\_Refs - 1 ) && interChange ) {

interChange = 0;

for ( int j = 0; j < No\_HabitatTable\_Refs - pass; j++ )

if ( m\_InsectTable[ j ] < m\_InsectTable[ j + 1 ] ) {

// set the flag

interChange = 1;

// The poly ref

hold1 = m\_HabitatTable\_PNum[ j ];

m\_HabitatTable\_PNum[ j ] = m\_HabitatTable\_PNum[ j + 1 ];

m\_HabitatTable\_PNum[ j + 1 ] = hold1;

// the size

hold1 = m\_HabitatTable\_Size[ j ];

m\_HabitatTable\_Size[ j ] = m\_HabitatTable\_Size[ j + 1 ];

m\_HabitatTable\_Size[ j + 1 ] = hold1;

// The insects

hold2 = m\_InsectTable[ j ];

m\_InsectTable[ j ] = m\_InsectTable[ j + 1 ];

m\_InsectTable[ j + 1 ] = ( int )hold2;

// The Pesticide

#ifdef \_\_PESTICIDE\_RA

hold2 = m\_PConcTable[ j ];

m\_PConcTable[ j ] = m\_PConcTable[ j + 1 ];

m\_PConcTable[ j + 1 ] = ( int )hold2;

#endif

}

}

}

|  |  |  |  |  |
| --- | --- | --- | --- | --- |
| virtual void Skylark\_Male::PesticideResponse | ( |  | ) |  |

protectedvirtual

Reimplemented from Skylark\_Base.

Referenced by EndStep().

|  |  |  |  |  |
| --- | --- | --- | --- | --- |
| void Skylark\_Male::ReEvaluateTerritory | ( |  | ) |  |

protected

Daily re-evaluation of territory.

The method is designed to keep the measure of territory quality up to date and avoids the need for the female to do this unless finding a nest position. The second usage is to provide the potential to reduce territory size should conditions improve. The reverse situation results in desertion by the female so is not handled here.

References skTTerritory::GetQuality(), skTTerritory::GetVirtualDiameter(), Skylark\_Base::m\_OurTerritories, m\_XFNestAcceptScore, Skylark\_Adult::MyTerritory, skTerritory\_struct::nqual, skTerritory\_struct::ref, SkTerritories::Split(), SkTerritories::Supply\_terr(), and skTerritory\_struct::validnest.

Referenced by BeginStep().

{

double qual = 0;

if (MyTerritory.validnest) {

qual = m\_OurTerritories->Supply\_terr( MyTerritory.ref )->GetQuality();

}

else qual=0;

if (qual>m\_XFNestAcceptScore) {

if (m\_OurTerritories->Supply\_terr( MyTerritory.ref )->GetVirtualDiameter()<50) return;

// He really has too much, so split it down into 2

m\_OurTerritories->Split( MyTerritory.ref );

qual = m\_OurTerritories->Supply\_terr( MyTerritory.ref )->GetQuality();

}

// Store the quality for today so the female can read it

MyTerritory.nqual = qual;

}

|  |  |  |  |  |
| --- | --- | --- | --- | --- |
| void Skylark\_Male::SensibleCopy | ( |  | ) |  |

|  |  |  |  |  |
| --- | --- | --- | --- | --- |
| bool Skylark\_Male::st\_Arriving | ( |  | ) |  |

protected

References Skylark\_Adult::GetBadWeather(), SkTerritories::GetTerritoriesByDistance(), Skylark\_Adult::m\_aTerrlist, Skylark\_Base::m\_Born\_x, Skylark\_Base::m\_Born\_y, Skylark\_Base::m\_OurPopulationManager, Skylark\_Adult::MyTerritory, Skylark\_Population\_Manager::TheSkylarkTerrs, skTerritory\_struct::x, and skTerritory\_struct::y.

Referenced by Step().

{

#ifdef \_\_CJTDebug\_5

if ( IsAlive() != 0x0DEADC0DE ) DEADCODEError();

#endif

if ( GetBadWeather() )

return false; // TransferToState(Flocking)

else {

m\_aTerrlist->clear();

int x = m\_Born\_x;

int y = m\_Born\_y;

if (this->MyTerritory.x>0) {

x = MyTerritory.x;

y = MyTerritory.y;

}

m\_OurPopulationManager->TheSkylarkTerrs->GetTerritoriesByDistance(x,y,m\_aTerrlist);

return true; // TransferToState(FindTerritory)

}

}

|  |  |  |  |  |
| --- | --- | --- | --- | --- |
| int Skylark\_Male::st\_AttractingAMate | ( |  | ) |  |

protected

Is called once each day. If a female comes along and pairs then she will trigger a move to FollowMate via Skylark\_Male::OnPairing

References g\_stopdate, HaveTerritory, TAnimal::m\_OurLandscape, Skylark\_Base::m\_OurTerritories, Skylark\_Adult::MyTerritory, skTerritory\_struct::ref, SkTerritories::RemoveMale(), and Landscape::SupplyDayInYear().

Referenced by Step().

{

#ifdef \_\_CJTDebug\_5

if ( IsAlive() != 0x0DEADC0DE ) DEADCODEError();

#endif

if ( m\_OurLandscape->SupplyDayInYear() > g\_stopdate ) {

m\_OurTerritories->RemoveMale( MyTerritory.ref );

MyTerritory.ref = -1; // No territory

HaveTerritory = false;

return 1; // TransferToState(Flocking)

}

else {

return 0; // Do nothing

}

}

|  |  |  |  |  |
| --- | --- | --- | --- | --- |
| int Skylark\_Male::st\_CaringForYoung | ( |  | ) |  |

protected

References Skylark\_Base::Age, FoodTripsPerDay, GetFood(), m\_Brood, m\_BroodSize, TAnimal::m\_OurLandscape, Skylark\_Base::m\_pcide\_conc, Skylark\_Base::m\_pesticide\_accumulation, Skylark\_Base::m\_Size, MaxFeedRain, Skylark\_Nestling::On\_FoodSupply(), random(), Skylark\_Adult::RemoveEM(), Landscape::SupplyDaylength(), and Landscape::SupplyRain().

Referenced by BeginStep().

{

#ifdef \_\_CJTDebug\_5

if ( IsAlive() != 0x0DEADC0DE ) DEADCODEError();

if ( m\_BroodSize < 1 )

DEADCODEError();

#endif

double spare; // spare food

int HungryChicks = m\_BroodSize;

int FeedingProbs[ 26 ];

double ChickFood[ 26 ];

#ifdef \_\_PESTICIDE\_RA\_CHICK

double ChickPoison[ 26 ];

#endif

if ( m\_Brood[ 0 ]->Age == 31 ) {

return 1; // Scare off chicks

}

// Check for bad weather

double RainToday = m\_OurLandscape->SupplyRain();

if (RainToday>MaxFeedRain) return 0; // No feeding today

// can only get food in daylight - dawn & dusk

int food\_time = ( int )( ( double )( m\_OurLandscape->SupplyDaylength() - 120 ));

double resources\_all = GetFood( food\_time ); // dw g insects

// remove metabolic requirements

double resources = RemoveEM( resources\_all );

if ( resources > 0 )

// Now give the food to the chicks

// The chance that a chick get a portion is proportional to their size

{

// Zero the feeding probabilities

for ( int i = 0; i < m\_BroodSize; i++ ) {

FeedingProbs[ i ] = 0;

ChickFood[ i ] = 0;

#ifdef \_\_PESTICIDE\_RA\_CHICK

ChickPoison[ i ] = 0;

#endif

}

resources /= FoodTripsPerDay; // Split into portions (food trips per day)

#ifdef \_\_PESTICIDE\_RA\_CHICK

double pcide\_remain = resources \* m\_pcide\_conc;

#endif

int BroodWeight = 0;

for ( int i = 0; i < m\_BroodSize; i++ ) {

// Add up brood weight

int weight = ( int )floor( m\_Brood[ i ]->m\_Size + 0.5 );

BroodWeight += weight;

for ( int j = i; j < m\_BroodSize; j++ ) FeedingProbs[ j ] += weight;

}

while ( ( resources > 0.01 ) && ( HungryChicks > 0 ) ) {

for ( int portion = 0; portion < FoodTripsPerDay; portion++ ) { // For each portion

int choose = random( BroodWeight );

for ( int i = 0; i < m\_BroodSize; i++ ) {

if ( choose < FeedingProbs[ i ] ) {

ChickFood[ i ] += resources; // store the food for that chick

#ifdef \_\_PESTICIDE\_RA\_CHICK

ChickPoison[ i ] += pcide\_remain;

#endif

break;

}

}

}

// Now give the food to the chicks

// Better do this than feed each a portion at a time, it is more efficient

// and stops problems with growth between portions

spare = 0;

for ( int i = 0; i < m\_BroodSize; i++ ) {

double extra = m\_Brood[ i ]->On\_FoodSupply( ChickFood[ i ] );

#ifdef \_\_PESTICIDE\_RA\_CHICK

// If we want a chick response to pesticide then the line below should be lit up, and the response

// specified in the PesticideResponse method

m\_Brood[ i ]->m\_pesticide\_accumulation += ChickPoison[i]\* ((ChickFood[ i ]-extra)/ChickFood[ i ]);

#endif

if ( extra > 0 ) // chick must be full

{

FeedingProbs[ i ] = -1; // no chance of food

HungryChicks--;

spare += extra;

}

}

resources = spare / FoodTripsPerDay;

}

}

return 0; // no change

}

|  |  |  |  |  |
| --- | --- | --- | --- | --- |
| void Skylark\_Male::st\_Dying | ( |  | ) |  |

protected

References TALMaSSObject::CurrentStateNo, HaveTerritory, m\_Brood, m\_BroodSize, Skylark\_Base::m\_CurrentSkState, Skylark\_Base::m\_OurTerritories, Skylark\_Female::MyMate, MyMate, Skylark\_Adult::MyTerritory, Skylark\_Nestling::OnDadDead(), Skylark\_Female::OnMateDying(), Skylark\_Adult::Paired, skTerritory\_struct::ref, SkTerritories::RemoveMale(), toss\_Destroy, and Landscape::Warn().

Referenced by Step().

{

#ifdef \_\_CJTDebug\_5

if ( IsAlive() != 0x0DEADC0DE ) DEADCODEError();

#endif

if ( MyMate ) {

if ( MyMate->MyMate != this ) {

g\_land->Warn( "Skylark\_Male::st\_Dying: DeBug1 ", NULL ); exit( 1 );

}

MyMate->OnMateDying();

}

if ( MyTerritory.ref != -1 ) m\_OurTerritories->RemoveMale( MyTerritory.ref );

MyTerritory.ref = -1; // No territory

HaveTerritory = false;

MyMate = NULL;

Paired = false;

if ( m\_BroodSize > 0 ) {

for ( int i = 0; i < m\_BroodSize; i++ ) {

m\_Brood[ i ]->OnDadDead();

}

}

CurrentStateNo = -1;

m\_CurrentSkState = toss\_Destroy;

}

|  |  |  |  |  |
| --- | --- | --- | --- | --- |
| int Skylark\_Male::st\_Emigrating | ( |  | ) |  |

protected

References Skylark\_Base::Age, April, February, g\_stopdate, GoodWeather, January, TAnimal::m\_OurLandscape, March, random(), Landscape::SupplyDayInYear(), and Landscape::SupplyTemp().

Referenced by Step().

{

#ifdef \_\_CJTDebug\_5

if ( IsAlive() != 0x0DEADC0DE ) DEADCODEError();

#endif

// Timing: Called once per day in the middle of a day

// In the emigration state GoodWeather must be 0 on first entry

int today = m\_OurLandscape->SupplyDayInYear();

if ( today >= g\_stopdate ) return 0; //don't come back this year

if ( m\_OurLandscape->SupplyTemp() > 0.0 ) GoodWeather++; else

GoodWeather = 0;

if ( GoodWeather >= 3 ) // Third day of good weather

{

int chance = random( 10000 ); // number between 0-99

if ( Age < 1 ) {

// below returning probability is divided by 30 as the number of days

if ( ( chance < 300 ) && ( today >= March ) ) return 1; else if ( today >= April ) return 1;

} else {

if ( ( chance < 7 ) && ( today >= January ) ) return 1;

else if ( ( chance < 33 ) && ( today >= February ) ) return 1;

else if ( ( chance < 300 ) & ( today >= March ) ) return 1;

else if ( today >= April ) return 1;

}

}

return 0; // return value of 0 do nothing, 1 go to immigration

}

|  |  |  |  |  |
| --- | --- | --- | --- | --- |
| int Skylark\_Male::st\_FindingTerritory | ( |  | ) |  |

protected

```
                      This option picks 10 territories at random and tests them.
```

This will happen if the bird arrives here without an m\_aTerrlist, or because it has exhausted its list of possible territories.

```
                     This option is for birds with a valid m_aTerrlist to search.
```

It takes the 10 nearest territories one at a time and tests to see if they are OK If so establish in one of them, otherwise wait until another day to test the next 10

References Skylark\_Base::Age, DefendTerritory(), Skylark\_Adult::m\_aTerrlist, TAnimal::m\_Location\_x, TAnimal::m\_Location\_y, m\_MyMinTerritoryQual, Skylark\_Base::m\_OurTerritories, Point::m\_x, Skylark\_Adult::MyTerritory, OnEvicted(), random(), skTerritory\_struct::ref, skTerritory\_struct::size, SkTerritories::Supply\_Owner(), SkTerritories::Supply\_quality(), SkTerritories::Supply\_size(), SkTerritories::Supply\_x(), SkTerritories::Supply\_y(), SkTerritories::SupplyIsNestValid(), SkTerritories::SupplyNoTerritories(), skTerritory\_struct::validnest, skTerritory\_struct::x, and skTerritory\_struct::y.

Referenced by Step().

{

Skylark\_Male \* TheOwner;

if (m\_aTerrlist->size()==0) {

#ifdef \_\_CJTDebug\_5

if ( IsAlive() != 0x0DEADC0DE ) DEADCODEError();

#endif

int Nterrs = m\_OurTerritories->SupplyNoTerritories();

int territories[ 10 ] [ 2 ];

// Select 10 territories

for ( int i = 0; i < 10; i++ ) {

territories[ i ] [ 0 ] = random( Nterrs );

TheOwner = m\_OurTerritories->Supply\_Owner( territories[ i ] [ 0 ] );

// If there is an owner

if ( TheOwner != NULL ) {

if ( TheOwner->DefendTerritory() >= Age ) {

territories[ i ] [ 1 ] = -1;

}

} else {

territories[ i ] [ 1 ] = m\_OurTerritories->Supply\_quality( territories[ i ] [ 0 ] );

}

}

int best\_qual = 0; // much too low

int best = -1;

for ( int i = 0; i < 10; i++ ) {

if ( territories[ i ] [ 1 ] > best\_qual ) {

best\_qual = territories[ i ] [ 1 ];

best = i;

}

}

if ( best\_qual > m\_MyMinTerritoryQual ) // Territory acceptable

{

// save ref number

TheOwner = m\_OurTerritories->Supply\_Owner( territories[ best ] [ 0 ] );

if ( TheOwner ) {

// Must send a get out message to the other bird

// if he is already breeding then fail

if ( TheOwner->OnEvicted() ) return 2; // fail

}

MyTerritory.ref = territories[ best ] [ 0 ];

// store territory data

MyTerritory.x = m\_OurTerritories->Supply\_x( MyTerritory.ref );

MyTerritory.y = m\_OurTerritories->Supply\_y( MyTerritory.ref );

MyTerritory.size = m\_OurTerritories->Supply\_size( MyTerritory.ref );

MyTerritory.validnest = m\_OurTerritories->SupplyIsNestValid(MyTerritory.ref);

return 1; //TransferToState(EstablishTerritory);

}

}

else {

for (int i=0; i<10 ; i++) {

int sz = int (m\_aTerrlist->size()-1);

int qual= 0 ;

if (sz >= 0) {

Point p = (\*m\_aTerrlist)[sz--];

m\_aTerrlist->pop\_back();

TheOwner = m\_OurTerritories->Supply\_Owner( p.m\_x );

// If there is an owner

if ( TheOwner != NULL ) {

if ( TheOwner->DefendTerritory() < Age ) {

qual = m\_OurTerritories->Supply\_quality( p.m\_x );

}

}

else {

qual = m\_OurTerritories->Supply\_quality( p.m\_x );

}

if ( qual > m\_MyMinTerritoryQual ) // Territory acceptable

{

if ( TheOwner ) {

// Must send a get out message to the other bird

// if he is already breeding then fail

if ( TheOwner->OnEvicted() ) return 2; // fail

}

MyTerritory.ref = p.m\_x;

// store territory data

MyTerritory.x = m\_OurTerritories->Supply\_x( MyTerritory.ref );

MyTerritory.y = m\_OurTerritories->Supply\_y( MyTerritory.ref );

MyTerritory.size = m\_OurTerritories->Supply\_size( MyTerritory.ref );

MyTerritory.validnest = m\_OurTerritories->SupplyIsNestValid(MyTerritory.ref);

return 1; //TransferToState(EstablishTerritory);

}

}

}

}

#ifdef FOR\_DEMONSTRATION

m\_Location\_x = 0;

m\_Location\_y = 0;

#endif

return 2; //TransferToState(Floating);

}

|  |  |  |  |  |
| --- | --- | --- | --- | --- |
| TTypesOfSkState Skylark\_Male::st\_Floating | ( |  | ) |  |

protected

References g\_stopdate, Skylark\_Adult::GetBadWeather(), TAnimal::m\_Location\_x, TAnimal::m\_Location\_y, TAnimal::m\_OurLandscape, Skylark\_Base::m\_OurPopulationManager, random(), Landscape::SupplyDayInYear(), Population\_Manager::SupplySimH(), Population\_Manager::SupplySimW(), toss\_MFindingTerritory, toss\_MFlocking, and toss\_MTempLeavingArea.

Referenced by BeginStep().

{

#ifdef \_\_CJTDebug\_5

if ( IsAlive() != 0x0DEADC0DE ) DEADCODEError();

#endif

if ( GetBadWeather() ) return toss\_MTempLeavingArea;

m\_Location\_x = random( m\_OurPopulationManager->SupplySimW() );

m\_Location\_y = random( m\_OurPopulationManager->SupplySimH() );

if ( m\_OurLandscape->SupplyDayInYear() > g\_stopdate ) {

return toss\_MFlocking;

} else {

#ifdef FOR\_DEMONSTRATION

m\_Location\_x = 0;

m\_Location\_y = 0;

#endif

return toss\_MFindingTerritory; //TransferToState(FindTerritory);

}

}

|  |  |  |  |  |
| --- | --- | --- | --- | --- |
| int Skylark\_Male::st\_Flocking | ( |  | ) |  |

protected

The bird is assumed to move around the area waiting until weather conditions are suitable to begin Emigration, depending upon the time of year. Only arrives here after breeding, so poor conditions will trigger a transition to Emigration. A transition to Emigration will also occur if the bird has not emigrated before October.

References Skylark\_Adult::GetBadWeather(), GoodWeather, TAnimal::m\_Location\_x, TAnimal::m\_Location\_y, TAnimal::m\_OurLandscape, October, September, and Landscape::SupplyDayInYear().

Referenced by Step().

{

#ifdef \_\_CJTDebug\_5

if ( IsAlive() != 0x0DEADC0DE ) DEADCODEError();

#endif

#ifdef FOR\_DEMONSTRATION

// TODO if we want it to look nice on the screen then the birds must flock

// this will not affect the rest of the simulation - just look better

// Can we be bothered????

// Just for now set the location to 0,0

m\_Location\_x = 0;

m\_Location\_y = 0;

#endif

if ( m\_OurLandscape->SupplyDayInYear() > October ) {

GoodWeather = 0;

return 1; //TransferToState(Emigration);

}

bool bad=GetBadWeather();

if ( (bad) && ( m\_OurLandscape->SupplyDayInYear() >= September )) return 1; //TransferToState(Emigration);

return 0; // Carry on

}

|  |  |  |  |  |
| --- | --- | --- | --- | --- |
| int Skylark\_Male::st\_FollowingMate | ( |  | ) |  |

protected

This is simply waiting for a message to say that either female is dead, female leaves or the eggs hatch. The male occupies the same location as the female. When the female's eggs hatch there is a transition to st\_CaringForYoung. If the female dies or abandons the territory, there is a transition to AttractingAMate. This method also has another function in that it sets up the food tables for the female, hence must be called before female activity starts.

References GetFood(), HaveTerritory, Skylark\_Base::m\_CurrentSkState, Skylark\_Base::m\_OurTerritories, Skylark\_Adult::MyTerritory, Skylark\_Adult::Paired, skTerritory\_struct::ref, SkTerritories::RemoveMale(), and toss\_MFloating.

Referenced by Step().

{

#ifdef \_\_CJTDebug\_5

if ( IsAlive() != 0x0DEADC0DE ) DEADCODEError();

#endif

if ( Paired ) {

GetFood( 0 ); //needs to set up the food tables for the female

}

else {

m\_CurrentSkState = toss\_MFloating;

if ( HaveTerritory ) {

HaveTerritory = false;

// Must de-register himself as territory owner

m\_OurTerritories->RemoveMale( MyTerritory.ref );

// Just to be sure we can set the ref to -1 = no territory

MyTerritory.ref = -1;

}

}

return 0;

}

|  |  |  |  |  |
| --- | --- | --- | --- | --- |
| bool Skylark\_Male::st\_Immigrating | ( |  | ) |  |

protected

An instantaneous state which determines the chance of migration mortality. If he does die then he has to inform any old mate that he has gone. If she is already paired with another bird then can just forget her. If not dying then the bird transitions to st\_Arrival.

References Skylark\_Base::Age, Skylark\_Adult::BSuccess, GetMigrationMortality(), m\_Brood, m\_BroodSize, MyMate, Skylark\_Female::OnMaleNeverComesBack(), Skylark\_Adult::Paired, and random().

Referenced by Step().

{

#ifdef \_\_CJTDebug\_5

if ( IsAlive() != 0x0DEADC0DE ) DEADCODEError();

#endif

if ( ( random( 100 ) < GetMigrationMortality() ) || Age > 5 ) {

if ( MyMate != NULL ) {

// Needs to be sure that she is not mated to someone else

if ( !MyMate->Paired ) {

MyMate->OnMaleNeverComesBack( this ); //stop a bogus on matedeath message

}

// In either case forget about mate - he no longer cares about this female

MyMate = NULL;

BSuccess = false;

#ifdef \_\_CJTDebug\_8

if ( m\_BroodSize != 0 )

int rubbish = 0;

for ( int i = 0; i < 6; i++ ) {

if ( m\_Brood[ i ] != NULL )

int rubbish = 0;

}

#endif

}

return false; // TransfertoState(Die)

} else

return true; // TransferToState(Arrival)

}

|  |  |  |  |  |
| --- | --- | --- | --- | --- |
| int Skylark\_Male::st\_ScaringOffChicks | ( |  | ) |  |

protected

References Skylark\_Adult::BSuccess, m\_Brood, m\_BroodSize, m\_firstPF, MyMate, Skylark\_Female::OnBreedingSuccess(), Skylark\_Nestling::OnDadDead(), and Skylark\_Female::OnStopFeedingChicks().

Referenced by Step().

{

#ifdef \_\_CJTDebug\_5

if ( IsAlive() != 0x0DEADC0DE ) DEADCODEError();

#endif

if ( m\_BroodSize > 0 ) {

for ( int i = 0; i < m\_BroodSize; i++ ) {

m\_Brood[ i ]->OnDadDead();

m\_Brood[ i ] = NULL;

}

m\_BroodSize = 0; // all chicks gone

MyMate->OnBreedingSuccess();

MyMate->OnStopFeedingChicks();

BSuccess = true;

}

m\_firstPF = false;

return 1; // TransferToState(FollowMate)

}

|  |  |  |  |  |
| --- | --- | --- | --- | --- |
| int Skylark\_Male::st\_TempLeavingArea | ( |  | ) |  |

protected

References Skylark\_Adult::GetBadWeather(), and GoodWeather.

Referenced by Step().

{

#ifdef \_\_CJTDebug\_5

if ( IsAlive() != 0x0DEADC0DE ) DEADCODEError();

#endif

// Timing - called once per day

// In the emigration state GoodWeather must be 0 on first entry

if ( !GetBadWeather() ) GoodWeather++;

else

GoodWeather = 0;

if ( GoodWeather >= 7 ) // 7 days of good weather

{

return 1; // comeback to the area

}

return 0; // return value of 0 do nothing, 1 go to Arrival

}

|  |  |  |  |  |  |
| --- | --- | --- | --- | --- | --- |
| void Skylark\_Male::Step | ( | void |  | ) |  |

virtual

Reimplemented from TAnimal.

References Skylark\_Base::Age, BroodAge, Calendar::Date(), EstablishingATerritory(), g\_date, HaveTerritory, m\_Brood, m\_BroodSize, Skylark\_Base::m\_CurrentSkState, TAnimal::m\_Location\_x, TAnimal::m\_Location\_y, Skylark\_Base::m\_OurPopulationManager, st\_Arriving(), st\_AttractingAMate(), st\_Dying(), st\_Emigrating(), st\_FindingTerritory(), st\_Flocking(), st\_FollowingMate(), st\_Immigrating(), st\_ScaringOffChicks(), st\_TempLeavingArea(), TALMaSSObject::StepDone, Population\_Manager::SupplyStateNames(), toss\_AttractingAMate, toss\_Destroy, toss\_FollowingMate, toss\_Initiation, toss\_MArriving, toss\_MDying, toss\_MEmigrating, toss\_MFindingTerritory, toss\_MFloating, toss\_MFlocking, toss\_MImmigrating, toss\_MTempLeavingArea, toss\_ScaringOffChicks, and Landscape::Warn().

{

#ifdef \_\_CJTDebug\_5

if ( IsAlive() != 0x0DEADC0DE ) DEADCODEError();

#endif

if ( StepDone || m\_CurrentSkState == toss\_Destroy ) return;

switch ( m\_CurrentSkState ) {

case toss\_Initiation: // Initial

m\_CurrentSkState = toss\_MFlocking;

break;

case toss\_MFlocking: // Flocking

switch ( st\_Flocking() ) {

case 3:

m\_CurrentSkState = toss\_MFindingTerritory; // -> find terr.

break;

case 2:

m\_CurrentSkState = toss\_MTempLeavingArea; // -> temp.leave

break;

case 1:

// use 0,0 as a default location

m\_Location\_x = 0;

m\_Location\_y = 0;

m\_CurrentSkState = toss\_MEmigrating; // -> Emigration

break;

case 0:

StepDone = true;

}

break;

case toss\_MArriving: // Arriving

if ( st\_Arriving() ) m\_CurrentSkState = toss\_MFindingTerritory; // -> find terr.

else {

m\_CurrentSkState = toss\_MTempLeavingArea;

}

StepDone = true;

break;

case toss\_MImmigrating: // Immigration - is instant

if ( st\_Immigrating() )

m\_CurrentSkState = toss\_MArriving; // -> arrival

else {

#ifdef TEST\_ISSUE\_DEATH\_WARRANT

printf( "Skylark\_Male::Step() : st\_Immigrating() : %d\n", ( int )g\_date->Date() );

#endif

m\_CurrentSkState = toss\_MDying; // -> die

}

break;

case toss\_MEmigrating: // Emigration

if ( st\_Emigrating() == 1 ) m\_CurrentSkState = toss\_MImmigrating; // -> immigr.

StepDone = true;

break;

case toss\_MTempLeavingArea: // Temp.leave.area

if ( st\_TempLeavingArea() ) m\_CurrentSkState = toss\_MArriving; // -> arrival

StepDone = true;

break;

case toss\_MFindingTerritory: // FindingTerritory

switch ( st\_FindingTerritory() ) {

case 1:

// Must do this now in case someone else gets this territory

EstablishingATerritory();

m\_CurrentSkState = toss\_AttractingAMate; // -> attract mate

break;

case 2:

HaveTerritory = false;

m\_CurrentSkState = toss\_MFloating; // -> floating

//m\_CurrentSkState = toss\_MTempLeavingArea;

StepDone = true;

break;

}

break;

case toss\_AttractingAMate: // Attract mate

if ( st\_AttractingAMate() ) {

m\_CurrentSkState = toss\_MFlocking; // -> flocking

}

StepDone = true;

break;

case toss\_FollowingMate: // FollowingMate

st\_FollowingMate();

StepDone = true;

break;

case toss\_ScaringOffChicks: // ScaringOffChicks - instant

if ( st\_ScaringOffChicks() ) m\_CurrentSkState = toss\_FollowingMate; // -> follow mate

break;

case toss\_MDying: // Dying

st\_Dying();

StepDone = true;

break;

default:

string st = m\_OurPopulationManager->SupplyStateNames( ( int )m\_CurrentSkState );

g\_land->Warn( "Skylark\_Male::Step: Unknown State", st.c\_str() );

exit( 0 );

break;

}

if ( m\_BroodSize ) BroodAge = m\_Brood[ 0 ]->Age; else

BroodAge = -1;

}

|  |  |  |  |  |
| --- | --- | --- | --- | --- |
| skTerritory\_struct Skylark\_Male::Supply\_Territory | ( |  | ) |  |

References Skylark\_Adult::MyTerritory.

Referenced by Skylark\_Female::EstablishTerritory(), and Skylark\_Female::st\_Finding\_Territory().

{

#ifdef \_\_CJTDebug\_5

if ( IsAlive() != 0xDEADC0DE )

DEADCODEError();

#endif

return MyTerritory;

}

|  |  |  |  |  |
| --- | --- | --- | --- | --- |
| double Skylark\_Male::Supply\_TerritoryQual | ( |  | ) |  |

inline

brief Provide todays territory quality

Referenced by Skylark\_Female::st\_BuildingUpResources(), Skylark\_Female::st\_MakingNest(), and Skylark\_Female::st\_StartingNewBrood().

{

return MyTerritory.nqual;

}

|  |  |  |  |  |  |
| --- | --- | --- | --- | --- | --- |
| int Skylark\_Male::SupplyBroodAge | ( | int | *n* | ) |  |

inline

Referenced by Skylark\_Female::FeedYoung(), and Skylark\_Female::st\_CaringForYoung().

{

return m\_Brood[n]->Age;

}

|  |  |  |  |  |
| --- | --- | --- | --- | --- |
| int Skylark\_Male::SupplyBroodSize | ( |  | ) |  |

inline

Referenced by Skylark\_Female::FeedYoung(), and Skylark\_Female::st\_GivingUpTerritory().

{

return m\_BroodSize;

}

|  |  |  |  |  |  |
| --- | --- | --- | --- | --- | --- |
| int Skylark\_Male::SupplyBroodWeight | ( | int | *n* | ) |  |

inline

Referenced by Skylark\_Female::FeedYoung().

{

return ( int )m\_Brood[n]->m\_Size;

}

|  |  |  |  |  |
| --- | --- | --- | --- | --- |
| Point Skylark\_Male::SupplyNestLoc | ( |  | ) |  |

inline

Referenced by Skylark\_Female::st\_MakingNest().

{

return m\_OurPopulationManager->TheSkylarkTerrs->SupplyNestPosition( MyTerritory.ref );

}

|  |  |  |  |  |
| --- | --- | --- | --- | --- |
| bool Skylark\_Male::SupplyNestValid | ( |  | ) |  |

inline

Referenced by Skylark\_Female::st\_MakingNest().

{

return m\_OurPopulationManager->TheSkylarkTerrs->SupplyIsNestValid( MyTerritory.ref);

}

|  |  |  |  |  |
| --- | --- | --- | --- | --- |
| int Skylark\_Male::SupplyNoHabitatRefs | ( |  | ) |  |

inline

{

return No\_HabitatTable\_Refs;

}

---

## Member Data Documentation

int Skylark\_Male::BroodAge

Referenced by Step().

int Skylark\_Male::GoodWeather

protected

Reimplemented from Skylark\_Adult.

Referenced by Skylark\_Male(), st\_Emigrating(), st\_Flocking(), and st\_TempLeavingArea().

bool Skylark\_Male::HaveTerritory

Referenced by EstablishingATerritory(), OnBroodDeath(), OnEvicted(), OnMateLeaving(), OnPreFledgelingMature(), OnReHouse(), Skylark\_Population\_Manager::ProbePOM(), Skylark\_Population\_Manager::ReHouse(), Skylark\_Male(), st\_AttractingAMate(), st\_Dying(), Skylark\_Female::st\_Finding\_Territory(), st\_FollowingMate(), Step(), and Skylark\_Population\_Manager::TheBreedingFemalesProbe().

Skylark\_Nestling\* Skylark\_Male::m\_Brood[26]

protected

Referenced by OnAddNestling(), OnAddPreFledgeling(), OnBroodDeath(), OnBroodDesertion(), OnMateDying(), OnMateLeaving(), OnNestlingDeath(), OnNestPredatation(), OnPreFledgelingDeath(), OnPreFledgelingMature(), Skylark\_Male(), st\_CaringForYoung(), st\_Dying(), st\_Immigrating(), st\_ScaringOffChicks(), and Step().

int Skylark\_Male::m\_BroodSize

protected

Referenced by OnAddPreFledgeling(), OnBroodDeath(), OnBroodDesertion(), OnMateDying(), OnMateLeaving(), OnNestlingDeath(), OnNestPredatation(), OnPreFledgelingDeath(), OnPreFledgelingMature(), Skylark\_Male(), st\_CaringForYoung(), st\_Dying(), st\_Immigrating(), st\_ScaringOffChicks(), and Step().

bool Skylark\_Male::m\_firstPF

protected

Referenced by OnAddPreFledgeling(), Skylark\_Male(), and st\_ScaringOffChicks().

vector<int> Skylark\_Male::m\_HabitatTable\_PNum

Referenced by ConstructAHabitatTable(), GetFood(), and OptimiseHabitatSearchingOrder().

vector<int> Skylark\_Male::m\_HabitatTable\_Size

Referenced by ConstructAHabitatTable(), GetFood(), and OptimiseHabitatSearchingOrder().

vector<double> Skylark\_Male::m\_InsectTable

Referenced by ConstructAHabitatTable(), GetFood(), and OptimiseHabitatSearchingOrder().

int Skylark\_Male::m\_MyMinTerritoryQual

protected

Referenced by OnMateLeaving(), Skylark\_Male(), and st\_FindingTerritory().

bool Skylark\_Male::m\_pesticide\_sprayed

protected

double Skylark\_Male::m\_XFNestAcceptScore

protected

Referenced by ReEvaluateTerritory(), and Skylark\_Male().

Skylark\_Female\* Skylark\_Male::MyMate

Referenced by Skylark\_Population\_Manager::Catastrophe(), OnAddPreFledgeling(), OnBroodDeath(), OnMateDying(), OnMateLeaving(), OnMateNeverComesBack(), OnPairing(), OnPreFledgelingMature(), OnReHouse(), Skylark\_Male(), Skylark\_Female::st\_Dying(), st\_Dying(), st\_Immigrating(), and st\_ScaringOffChicks().

int Skylark\_Male::No\_HabitatTable\_Refs

protected

Referenced by ConstructAHabitatTable(), GetFood(), and OptimiseHabitatSearchingOrder().

---

The documentation for this class was generated from the following files:

- skylarks\_all.h
- skylarks\_all.cpp


- Skylark\_Male
- Generated on Thu Jan 10 2013 13:15:38 for ALMaSS Skylark ODdox by
   1.8.1.1
